# Can male Eurasian jays disengage from their own current desire to feed the female what she wants?

**DOI:** 10.1098/rsbl.2014.0042

**Published:** 2014-03

**Authors:** Ljerka Ostojić, Edward W. Legg, Rachael C. Shaw, Lucy G. Cheke, Michael Mendl, Nicola S. Clayton

**Affiliations:** 1 Department of Psychology, University of Cambridge, Downing Street, Cambridge CB2 3EB, UK; 2 Centre for Behavioural Biology, School of Veterinary Science, University of Bristol, Langford House, Langford, Bristol BS40 5DU, UK

**Keywords:** Eurasian jay, corvid, food-sharing, specific satiety, desire-state attribution

## Abstract

Humans' predictions of another person's behaviour are regularly influenced by what they themselves might know or want. In a previous study, we found that male Eurasian jays (*Garrulus glandarius*) could cater for their female partner's current desire when sharing food with her. Here, we tested the extent to which the males' decisions are influenced by their own current desire. When the males' and female's desires matched, males correctly shared the food that was desired by both. When the female's desire differed from their own, the males' decisions were not entirely driven by their own desires, suggesting that males also took the female's desire into account. Thus, the male jays' decisions about their mates' desires are partially biased by their own desire and might be based upon similar processes as those found in humans.

## 1. Introduction

State-attribution is the ability to ascribe to another individual an internal life like one's own and understand that internal, psychological states govern the other's actions. Humans are thought to rely on their own experience to infer that a similar experience might influence another's behaviour [1]. However, to correctly attribute an internal state to another, we need to inhibit our own current state. Children develop the ability to respond to others' desires that match their own before developing the ability to respond to conflicting desires [2,3]. For example, when asked by a protagonist to hand them either broccoli or biscuits, both 14- and 18-month-old infants, both of whom always prefer biscuits over broccoli, responded correctly by giving the biscuits when the protagonist's desire matched their own. However, when the protagonist's desire conflicted with their own only the 18-month olds could respond appropriately by handing the protagonist the broccoli [2]. Importantly, when desires are matched, children can pass the task by responding to their own rather than the protagonist's desire. Only conflicting desires tasks, in which participants need to inhibit their own current state, can provide evidence for state-attribution.

When predicting the behaviour of others, adults are also regularly biased by their own internal states [1]. Individuals with access to specialized information about an event tend to overestimate the knowledge of others that are uninformed about the event [4]. Similarly, individuals who experience thirst after heavy exercise are more likely to attribute thirst to protagonist hikers in a story than individuals who have not yet exercised [1].

In a recent study, we found that a member of the corvid family, the Eurasian jay (*Garrulus glandarius*)*,* can attribute desires to another individual. We used the jays' courtship behaviour of food-sharing to investigate whether male jays respond to their partner's current desire [5]. After seeing the female being pre-fed on a particular food (resulting in her decreased desire for that food, termed ‘specific satiety’), the males correctly responded by sharing less of that food. Importantly, when males did not see the female during pre-feeding and her behaviour at the time of sharing was the only information available, they did not modulate their sharing pattern appropriately. Thus, males were not simply using ‘stimulus-bound behaviour reading’ [6]; their sharing pattern was not a response to the female's behaviour during the test phase that could have signalled what food she wanted to be fed [7]. Instead, males required the information about what *caused* the change in the female's desire for a food (i.e. eating it to satiety), suggesting that their sharing behaviour might be based on attributing an internal state to the female. Finally, it was shown that the males' own motivation was not influenced by what the female had eaten; when there was no possibility to share with the female, the males' choices for themselves did not follow the pattern shown in their sharing behaviour [5]. Thus, the males' sharing behaviour was a response to their partner's desire, rather than their own, suggesting that the males' decisions about what to choose for themselves and what to choose for another individual are distinct [7]. In the original study, the males' desire was neutral towards the test foods and held constant across trials by always pre-feeding them maintenance diet (MD). It is not yet known to what extent males can disengage from their own current desire-state and respond to the female's desire when they themselves experience different desires for the test foods.

The aim of this study is to test how the males' sharing behaviour is affected when they experience specific satiety for one of the two test foods, and the female's desire is manipulated to be either *matched* (by being pre-fed the same food), *conflicting* (by being pre-fed the other food) or *neutral* towards the test foods (by being pre-fed MD). If the males' food-sharing decisions are entirely driven by their own desire, their sharing pattern should be in line with their own specific satiety in all conditions. If the males can entirely disengage from their own desires, their sharing pattern should be in line with their own specific satiety only in the *matched* condition, while in the other two conditions they should share food solely according to what the female wants. Finally if, like humans, the males' decisions are partially biased by their own desire, their response to the female's desire should be better in the *matched* condition than when the female's desire differs from their own.

## 2. Material and methods

### (a) Subjects

Nine male–female pairs from two colonies (colony 1: *n* = 4 pairs, colony 2: *n* = 5 pairs) were tested during the breeding seasons (March to June) in 2012 and 2013, which is the only time when jays share food. Seven pairs had participated in the original food-sharing study [5]. The two new pairs first participated in a specific satiety experiment and the original seen condition of the food-sharing experiment (for details of procedure, see [5]), which ensured that they had specific satiety and exhibited the original effect. Birds were housed in outdoor aviaries (20 × 6 × 3 m) and tested in indoor compartments (2 × 1 × 2 m). Birds were fed an MD of soaked dog biscuits, cheese, seeds, nuts and fruit and had ad libitum access to water.

### (b) Procedure

**Figure 1.**
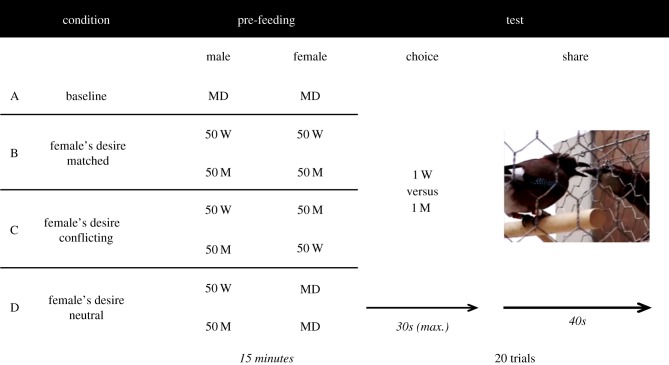
Outline of experimental procedures. ‘Pre-feeding’ and ‘test’ columns depict the type and quantity of food given to the birds: maintenance diet (MD), wax moth larvae (W) or mealworm larvae (M), with duration of the phases given in italics. (Online version in colour.)

To ensure that the birds were mildly hungry and thus motivated to eat the pre-fed food, MD was removed approximately 2 h before testing. Pairs were tested once a day. During testing, females and males were placed in adjacent compartments joined by a mesh window. All trials consisted of a pre-feeding and a test phase. In a baseline trial, the birds were pre-fed a handful of MD (neutral desire towards the test foods) to assess the males' general preference for sharing the test foods. In the test trials, the male always experienced specific satiety for the test foods (by being pre-fed wax moth larvae (W) or mealworm beetle larvae (M)), while the female's desire was either *neutral* (pre-fed MD), *matched* (pre-fed same food as male) or *conflicting* (pre-fed the other food; figure 1). The order of the *matched* desires and *conflicting* desires conditions was counterbalanced across pairs as was the order, in which the male was pre-fed W and M. Birds were tested on the *neutral* desires condition last. In all test trials, both foods were present (either on the floor or an elevated platform in front of the female's compartment) and visible to the males during pre-feeding to control for visual and odour cues. During the pre-feeding phase, a transparent Perspex screen covered the mesh window to prevent the males from sharing food. At the end of pre-feeding, all food and the Perspex screen were removed.

In the test phase of each test condition, males were given 20 choices of a single W and M. For six males, the experimenter held one larva in each hand against the mesh of the compartment. For three males who were not tame enough for this procedure (Ayton, Dublin, Lisbon), the choices were presented on a platform inside the compartment. The position of the foods was pseudo-randomized with no food appearing on the same side on more than two consecutive trials. If no choice was made within 30 s, the foods were removed. Each opportunity to make a choice was followed by 40 s, in which males could either eat, cache or feed the food to the female through the mesh window.

### (c) Analysis

Data were live scored by LO for colony 1 and RCS (two pairs) and EWL (three pairs) for colony 2. Twenty per cent of trials were video scored by a naive rater and compared to live scores (items chosen: Cohen's *κ* = 1; items shared: Cohen's *κ* = 0.824).

The results from the baseline show that males preferred to choose and share W over M (table 1). To investigate how the different pre-feeding trials affected this preference, for each trial, we calculated the number of W minus the number of M shared or chosen: (W–M). All graphs show the difference between these values in a test trial (male pre-fed W and pre-fed M) and the baseline (male pre-fed MD): [(W–M)_male pre-fed W or M_ – (W–M)_male pre-fed MD_]. This ensured that inter-individual variation in the amount of food shared as well as in general food preferences were taken into account [5]. Error bars represent standard errors of the mean calculated using the Cousineau method (2005), which controls for between-subject variation [8]. The original analysis used by Ostojić *et al*. (2013) measured the proportion of W out of total number of items shared [5]. Unlike proportional data, the current measurement takes into account trials in which males shared 0W and 0M and data from males whose preference for sharing W was so strong that they shared only W throughout all trials. If the males' choice and sharing pattern are in accordance with their own desire, their preference for W over M relative to the baseline is expected to be lower when males were pre-fed W than when pre-fed M.

**Table 1.** Food items chosen and shared.

condition	food pre-fed		Ayton		Caracas		Dublin		Hoy		Lima		Lisbon		Pendleton		Romero		Wilson	
	male	female	W	M	W	M	W	M	W	M	W	M	W	M	W	M	W	M	W	M
The males' choices. The food pre-fed column refers to the food type (MD, W or M) fed to each individual during the pre-feeding phase of the experiment																				
baseline	MD	MD	13	5	13	2	17	2	14	6	17	3	18	1	17	2	11	9	13	4
*matched*	W	W	2	0	4	9	5	3	12	4	10	8	0	4	4	3	3	3	15	4
	M	M	15	0	15	4	3	1	16	3	14	2	8	1	2	0	11	9	19	1
*conflicting*	W	M	5	0	1	19	11	6	10	10	16	4	0	2	8	9	4	3	15	0
	M	W	13	3	4	11	10	5	19	1	20	0	0	1	4	2	7	3	17	3
*neutral*	W	MD	4	5	9	4	3	0	3	1	16	4	3	1	13	5	7	12	2	2
	M	MD	7	0	11	0	10	0	17	3	14	6	8	0	19	1	5	3	5	1
The males’ food sharing pattern. The food pre-fed column refers to the food type (MD, W or M) fed to each individual during the pre-feeding phase of the experiment																				
baseline	MD	MD	2	0	8	1	3	0	5	0	8	1	5	0	5	0	9	3	4	0
*matched*	W	W	0	0	0	0	0	1	1	0	0	0	0	0	0	0	0	1	0	0
	M	M	8	0	4	0	0	0	1	0	0	0	4	0	2	0	5	0	3	0
*conflicting*	W	M	6	0	0	0	3	0	11	0	0	0	0	0	1	0	1	3	6	0
	M	W	0	0	1	5	3	0	2	0	0	0	0	0	2	1	0	0	5	1
*neutral*	W	MD	1	0	6	0	1	0	4	0	6	0	1	0	8	0	5	3	1	0
	M	MD	2	0	4	0	4	0	6	0	4	1	4	0	9	0	4	0	1	0

Two males (Ayton on one trial, Hoy on two trials) shared with the female not only the items chosen during the test phase but also one item that they kept from the pre-feeding phase. These items have to be considered as part of the males' decision as to what to feed to the female and were thus included in the analysis.

All analyses were planned contrasts, performed using exact permutation tests [9]. All tests were two-tailed. Alpha was set at 0.05. *P*-values between 0.05 and 0.10 were interpreted as trends.

## 3. Results

### (a) Items shared

**Figure 2.**
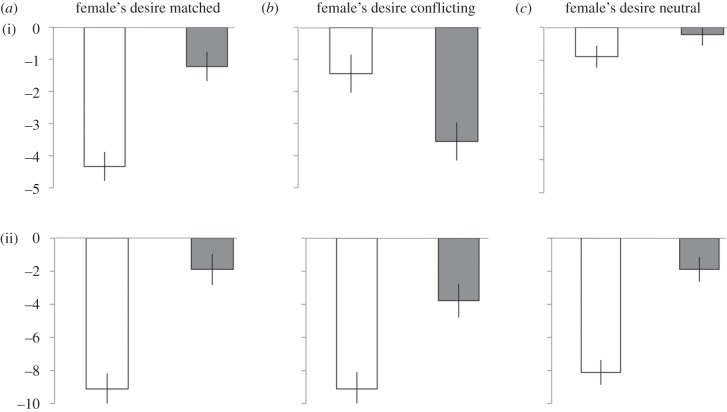
Mean (±s.e.m.) difference in the number of W minus the number M (i) shared or (ii) chosen in a test trial and the baseline when the female's desire was (*a*) matched, (*b*) conflicting or (*c*) neutral (white bars denote males pre-fed W; grey bars denote males pre-fed M).

In the *matched* condition, the males' sharing pattern was in line with their own specific satiety; the preference for W over M relative to the baseline was lower when males were pre-fed W than when pre-fed M (*n* = 9, *Z* = − 2.32, *p* = 0.02; figure 2*a*(i)). Furthermore, the males' sharing pattern was not solely driven by their own desire when the female had a *conflicting* (*n* = 9, *Z* = 1.60, *p* = 0.19; figure 2*b*(i)) or *neutral* desire (*n* = 9, *Z* = − 1, *p* = 0.43, figure 2*c*(i)). In the *conflicting* condition, the lack of a difference between the test trials means that here males could also not entirely disengage from their own desire to perfectly cater for what the female wanted. However, the males' sharing pattern differed between when the female's desire *matched* and when it was *conflicting* (*n* = 9, *Z* = 2.34, *p* = 0.008; figure 2*a*(i) versus figure 2*b*(i)). Furthermore, there were trends for the males' sharing pattern to differ when the female's desire was *matched* and when it was *neutral* (*n* = 9, *Z* = 1.96, *p* = 0.055; figure 2*a*(1) versus figure 2*c*(i)) as well as when the female's desire was *conflicting* and when it was *neutral* (*n* = 9, *Z* = − 1.763, *p* = 0.08; figure 2*b*(i) versus figure 2*c*(i)). As the males' desire was manipulated in the same way across all three conditions, these differences in the sharing pattern can be attributed to the different desires experienced by the female.

### (b) Items chosen

The males' choices were in line with their own desire in all three conditions; the preference for W over M relative to the baseline was lower when males were pre-fed W than when pre-fed M (figure 2*a*(ii)–*c*(ii), *n* = 9; *neutral*: *Z* = −2.48, *p* = 0.012; *matched*: *Z* = −2.41, *p* = 0.008, *conflicting*: *Z* = −2.04, *p* = 0.040). In addition, the males' choice pattern did not differ between the different conditions (*n* = 9; *neutral* versus *matched*: *Z* = 0.38, *p* = 0.731; *neutral* versus *conflicting*: *Z* = −0.41, *p* = 0.734; *matched* versus *conflicting*: *Z* = 0.82, *p* = 0.477).

## 4. Discussion

After watching their female partner being pre-fed different foods and while experiencing their own specific satiety for the test foods, the males' food choices were based upon their own desire. By contrast, the males' sharing pattern was only partially influenced by their own specific satiety. In the *matched* condition, males fed the female in line with her specific satiety. However, this response could have been based entirely on the males' own desire. Critically, when the female's desire differed from the males' desire (*conflicting* and *neutral*), the males' sharing pattern was not solely influenced by their own specific satiety, indicating that they could take the female's desire into account.

Our results show that—just like human adults and children in similar situations [1–4]—the male jays' decisions for another individual are biased, but not entirely driven, by their own desire-state. This bias in humans is thought to arise because we use the experience of our own internal states to ascribe similar states to others. The present findings suggest that this link between one's own experience and the response to another's internal state might not be limited to humans but that a similar process might also govern non-human state-attribution.

## Notes

The experiments were approved by the University of Cambridge Ethics Review Process.

## Data accessibility

The raw data are presented in table 1.
